# A Reproducible Sequence-Level Strategy to Enhance Peptide Immunogenicity While Preserving Wild-Type Epitope Recognition

**DOI:** 10.3390/antib14040106

**Published:** 2025-12-12

**Authors:** Chia-Hung Chen, Yu-Chi Chiu, Kai-Yao Huang, Hsiao-Hsuan Huang, Ta-Wei Kuo, Yu-Chi Liu, Hui-Ju Kao, Chen-Lin Yu, Shun-Long Weng, Kuang-Wen Liao

**Affiliations:** 1 Department of Medical Research, Hsinchu MacKay Memorial Hospital, Hsinchu 30071, Taiwan; steven.l862@mmh.org.tw (C.-H.C.); kyh.l765@mmh.org.tw (K.-Y.H.); hjk.l908@mmh.org.tw (H.-J.K.); l204.l204@mmh.org.tw (C.-L.Y.); 2 Department of Medical Research, Hsinchu Municipal MacKay Children’s Hospital, Hsinchu 30068, Taiwan; 3 Department of Internal Medicine, Taoyuan General Hospital, Ministry of Health and Welfare, Taoyuan 33004, Taiwan; newchiuyuchi@gmail.com; 4 Department of Medicine, MacKay Medical College, No. 46, Sec. 3, Zhongzheng Rd., New Taipei 25245, Taiwan; 5 Institute of Biomedical Sciences, MacKay Medical College, New Taipei 25245, Taiwan; 6 Department of Biological Science and Technology, College of Engineering Bioscience, National Yang Ming Chiao Tung University, No. 75 Po-Ai Street, Hsinchu 30068, Taiwan; hsuan.bt10@nycu.edu.tw (H.-H.H.); jesuskuo32@gmail.com (T.-W.K.); swatchnctu@yahoo.com.tw (Y.-C.L.); 7 Department of Obstetrics and Gynecology, Hsinchu MacKay Memorial Hospital, Hsinchu 30071, Taiwan; 8 Department of Nursing, Mackay Junior College of Medicine, Nursing and Management, Taipei 11260, Taiwan; 9 Department of Obstetrics and Gynecology, Hsinchu Municipal MacKay Children’s Hospital, Hsinchu 30068, Taiwan; 10 Institute of Molecular Medicine and Bioengineering, College of Engineering Bioscience, National Yang Ming Chiao Tung University, Hsinchu 30068, Taiwan; 11 Center for Intelligent Drug Systems and Smart Bio-Devices (IDS^2^B), National Yang Ming Chiao Tung University, Hsinchu 30068, Taiwan; 12 Department of Biotechnology and Bioindustry Sciences, National Cheng Kung University, Tainan 70101, Taiwan; 13 Graduate Institute of Medicine, College of Medicine, Kaohsiung Medical University, Kaohsiung 80708, Taiwan; 14 School of Dentistry, Kaohsiung Medical University, Kaohsiung 80708, Taiwan; 15 Drug Development and Value Creation Research Center, Kaohsiung Medical University, Kaohsiung 80708, Taiwan

**Keywords:** peptide immunogenicity, sequence-level antigen engineering, conservative (heteroclitic) mutagenesis, cross-reactive antibodies

## Abstract

**Background:** Short peptide epitopes are valuable for mechanistic studies, yet their intrinsic low immunogenicity and lack of commercial antibodies hinder rapid antibody generation. **Methods:** We developed a reproducible, sequence-level workflow combining cross-species/structural triage, independent MHC-I/II prioritization, and conservative heteroclitic-style substitutions to enhance predicted MHC affinity while preserving native epitope features. Using visfatin as a model, two optimized fragments were conjugated to KLH and tested in mice for antibody titers, isotype profiles, and binding kinetics. **Results:** Mutant peptides improved MHC-binding prediction, elicited stronger antibody titers, and promoted isotype maturation (increased IgG1). Importantly, antibodies maintained measurable binding to wild-type sequences, indicating preserved cross-recognition. Similar effects were reproduced with additional antigens. **Conclusions:** This proof-of-concept study, based on small exploratory mouse cohorts (*n* = 3 per group), demonstrates that strategic, minimal sequence edits can significantly enhance peptide immunogenicity while preserving native epitope recognition. This streamlined workflow provides a low-barrier route to generate epitope-directed antibodies when commercial reagents are unavailable.

## 1. Introduction

Short peptide epitopes are indispensable tools for probing protein function—whether in Western blotting, epitope blocking, or pathway dissection [1]. However, the most informative regions often lack commercially available antibodies, creating a persistent bottleneck for mechanistic studies [2]. While whole proteins are inherently more immunogenic and commonly used for hybridoma-based antibody generation, this approach is time-consuming and inefficient when the goal is to obtain antibodies targeting a specific functional fragment. Screening large panels of hybridoma clones to identify those recognizing the correct epitope requires multiple selection rounds, and in many cases, suitable clones cannot be recovered at all [3,4,5]. Conceptually similar challenges have been reported for haptens and other small antigens, where subtle structural modifications or changes in hapten density have been shown to enhance immunogenicity while preserving native antigenic recognition [6,7].

To circumvent these limitations, researchers often immunize animals with short, sequence-defined peptide antigens representing the region of interest. However, short peptides typically exhibit low intrinsic immunogenicity and high sequence conservation across species or paralogs, resulting in weak immune responses and limited specificity [2,4,8,9]. Conventional strategies to enhance peptide immunogenicity—such as carrier conjugation (e.g., KLH, OVA, BSA), adjuvant-rich formulations, or the use of long or overlapping peptides—can increase antibody titers but often skew immune responses toward carrier-dominant epitopes, complicate formulation, or fail to address intrinsic sequence limitations [10,11,12]. These approaches primarily amplify immune stimulation extrinsically, without improving the peptide’s inherent immunogenic potential. Consequently, such constraints delay experimental progress and hinder the development of diagnostic or therapeutic tools that require rapid and precise antibody generation.

To overcome these intrinsic limitations, rational sequence-level modifications have emerged as a promising alternative. Conservative heteroclitic substitution design, which introduces minimal physicochemically similar substitutions to enhance immunogenicity, has been successfully applied in T-cell epitope contexts such as tumor-associated and viral antigens, where rational substitutions improve MHC binding while preserving recognition of native sequences [13,14]. However, systematic applications of this concept to B-cell or antibody epitopes remain comparatively scarce, leaving a methodological gap in rational antibody generation against short peptides.

To address this unmet need, we developed a compact and reproducible sequence-level workflow that integrates three key steps: (i) triaging candidate segments by cross-species homology and structural context to enrich for surface-exposed loops while excluding buried or PTM-prone regions; (ii) independently prioritizing peptides for MHC class I and class II presentation; and (iii) applying conservative heteroclitic substitutions to enhance predicted MHC binding while maintaining native epitope integrity. Heteroclitic-style conservative substitutions refer to rational amino-acid replacements that preserve physicochemical similarity while enhancing MHC engagement. Here, unlike classical heteroclitic peptides designed for T-cell activation, our application focuses on maintaining B-cell epitope architecture while improving immunogenicity. Using visfatin as a model antigen, we demonstrate that KLH-conjugated, rationally optimized peptides elicit stronger antibody responses, promote isotype maturation (notably increased IgG1), and retain recognition of wild-type sequences with improved binding affinity. Keyhole limpet hemocyanin (KLH) is a high-molecular-weight, highly immunogenic carrier protein widely used to enhance antibody responses against small peptide antigens. Moreover, spatially arranged double substitutions reveal how mutation positioning modulates recognition, while extending the workflow to additional antigens validates its generalizability.

Together, this study introduces a practical and broadly applicable sequence-level strategy to enhance peptide immunogenicity through minimal, conservative substitutions while preserving native-epitope recognition—providing a low-barrier and reproducible framework for generating application-ready, epitope-directed antibodies when commercial reagents are unavailable.

## 2. Materials and Methods

### 2.1. Peptide Candidate Selection and Computational Triage

**Figure 1:**
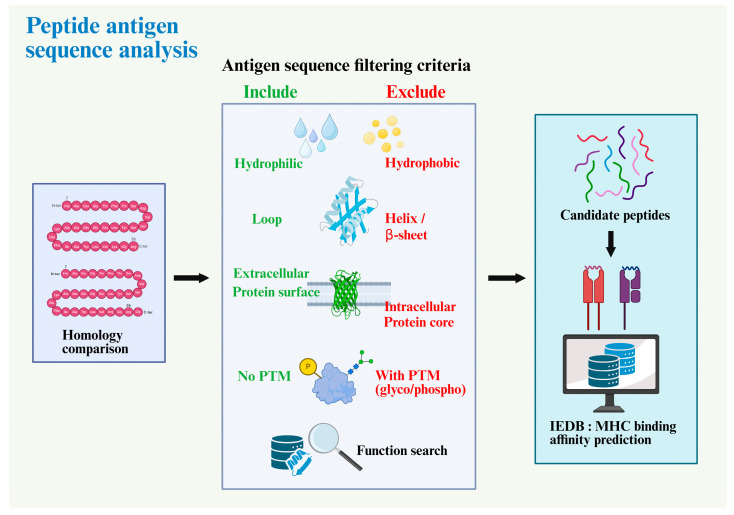
**Workflow for peptide antigen sequence analysis and prioritization.** Schematic of the screening pipeline used to nominate peptide antigens. (**Left**): cross-species homology comparison is performed to avoid regions highly similar to host proteins. (**Middle**): sequence/structure filtering criteria—Include: hydrophilic residues, loop propensity, extracellular/protein-surface localization, and absence of predicted post-translational modifications (PTMs); Exclude: hydrophobic segments, α-helix/β-sheet cores, intracellular/buried protein-core regions, and sites with predicted PTMs (glycosylation/phosphorylation). Functional annotation is queried to retain fragments with relevant biological context. (**Right**): retained candidate peptides are submitted to the IEDB platform for MHC binding affinity prediction (class I/II), providing ranked lists for subsequent mutagenesis and experimental validation. Abbreviations: PTM, post-translational modification; IEDB, Immune Epitope Database; MHC, major histocompatibility complex.

**Figure 2:**
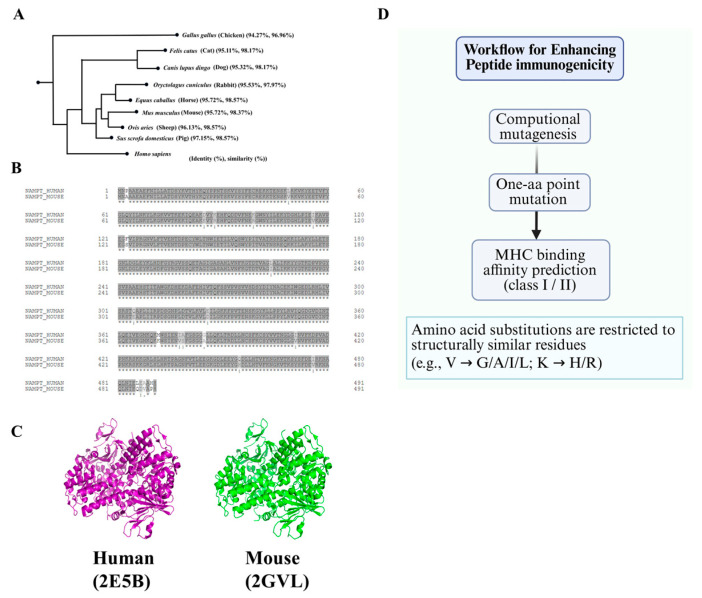
**Cross-species conservation of visfatin and sequence-level design workflow for peptide immunogenicity enhancement.** (**A**) Phylogenetic analysis of visfatin/NAMPT across representative vertebrates showing pairwise identity and similarity (%) relative to human, indicating strong evolutionary conservation. (**B**) Human–mouse visfatin protein alignment. Shaded blocks denote conserved residues; gaps or highlighted residues indicate local divergence used to nominate candidate peptide regions. ‘*’ indicates fully conserved residues; ‘:’ denotes strongly similar residues; and ‘.’ represents weakly similar residues. (**C**) Crystal structures of human (PDB: 2E5B) and mouse (PDB: 2GVL) visfatin illustrating overall structural similarity and surface accessibility of loop regions considered for epitope selection. (**D**) Sequence-level optimization pipeline. Candidate peptides undergo computational mutagenesis with single-amino-acid substitutions restricted to physicochemically similar residues (e.g., V→G/A/I/L; K→H/R). Variants are prioritized by IEDB MHC class I/II binding predictions for subsequent experimental validation.

To enable reproducible peptide selection, we implemented a predefined triage workflow combining cross-species filtering, structural annotation, and MHC-binding prediction (Figure 1). Full-length human and mouse visfatin/NAMPT sequences were retrieved from UniProt (https://www.uniprot.org/ (accessed on 18 February 2022)) and aligned using Clustal Omega (https://www.ebi.ac.uk/Tools/msa/clustalo/ (accessed on 18 February 2022)). Regions with <95% identity were considered locally divergent and retained for further screening (Figure 2).

Candidate segments were next evaluated for structural suitability using the human (PDB: 2E5B) and mouse (PDB: 2GVL) visfatin crystal structures. Regions were included if they demonstrated: (i) loop propensity or flexible secondary structure, (ii) surface exposure (solvent-accessible surface area > 25% of residue max ASA), and (iii) absence of predicted PTMs, including glycosylation (NetNGlyc/NetOGlyc) or phosphorylation (NetPhos).

Segments were excluded if located within buried cores, α/β structural elements, or predicted transmembrane regions.

For MHC-binding prioritization, each candidate sequence was submitted to the IEDB MHC-I and MHC-II prediction tools using the BALB/c haplotypes H2-Kd (class I) and H2-IEd (class II). Variants were ranked by percentile binding score, with ≤20% considered acceptable and ≤10% prioritized for mutagenesis. Conservative substitutions were generated by restricting mutations to physicochemically similar residues (e.g., F→Y, T→M), selected if they improved predicted MHC affinity without altering charge class or polarity. In this study, mutant peptides refer to peptide variants engineered with conservative substitutions, meaning minimal residue changes restricted to physicochemically similar amino acids to preserve native epitope geometry.

Two optimized peptides (one class-I-prioritized, one class-II-prioritized) were advanced for synthesis and in vivo validation.

### 2.2. KLH-Peptide Antigen Preparation

Synthetic peptides were obtained from Angene Biotech (Taipei, Taiwan, China). Lyophilized peptides were dissolved in sterile deionized water to prepare stock solutions (10 mg/mL) and stored at −20 °C until use. On each immunization day, an aliquot containing 300 µg of peptide stock (sufficient for three mice at 100 µg peptide per mouse) was transferred into phosphate-buffered saline (PBS, pH 7.2). Sulfo-SMCC (Sigma, St. Louis, MO, USA) was then added at a 1:10 molar ratio (peptide/sulfo-SMCC) and incubated for 30 min at room temperature to activate the crosslinker. The activated peptide was subsequently conjugated to KLH at an 80:1 molar ratio (peptide/KLH) and used immediately for immunization without further purification.

### 2.3. Animal Welfare and Monitoring

Mice were monitored daily for general health status, activity, posture, grooming, injection-site reactions, and weight loss (>20% as a humane endpoint). All procedures were performed in accordance with institutional guidelines to minimize pain and distress. Mice were housed under standard husbandry conditions (12 h light/dark cycle, controlled temperature and humidity, and libitum access to food and water). No adverse events were observed during the study.

### 2.4. Immunization

Female BALB/c mice (4–5 weeks old; *n* = 3 per group) were immunized subcutaneously on day 0 with freshly prepared KLH–peptide conjugates corresponding to 100 µg of peptide per mouse. Each dose was emulsified with 50 µL of complete Freund’s adjuvant (CFA; Sigma, St. Louis, MO, USA) and adjusted to a final injection volume of 100 µL. Booster injections were administered on days 14, 28, and 42, using the same amount of KLH–peptide antigen emulsified with 50 µL of incomplete Freund’s adjuvant (IFA; Sigma, St. Louis, MO, USA), again in a total volume of 100 µL. Control mice received KLH-conjugated wild-type peptide antigens prepared using the same conjugation procedure. Blood samples were collected at baseline and on days 14, 28, 42, and 56 post-immunization for serum preparation. Peptide variant identifiers (e.g., V1-1, V2-1) denote sequence variants, whereas individual mice within each group are referred to as mice 1–3.

### 2.5. Antibody Titer and Isotype Distribution

Antibody titers were determined by ELISA. Briefly, 96-well plates were coated overnight at 4 °C with 10 µg/mL peptide antigen in carbonate buffer (pH 9.6). After blocking with 2% (*w*/*v*) skim milk in PBS, serial serum dilutions were added and incubated for 1 h at room temperature. Bound antibodies were detected using HRP-conjugated anti-mouse IgG (Arigo, Zhubei, Taiwan, China), and absorbance was measured at 450 nm. Isotype distribution (IgM, IgA, IgG1, IgG2a, IgG2b) was determined using isotype-specific secondary antibodies (Arigo, Zhubei, Taiwan, China) according to the manufacturer’s instructions.

### 2.6. Monoclonal Antibody Purification

Monoclonal antibodies were purified from hybridoma culture supernatants using protein G affinity chromatography (GE17-0618-02, Merck, Darmstadt, Germany). They were subsequently dialyzed against 1× PBS with buffer replacement twice within 24 h to remove excess salts. Purified antibody concentrations were determined by spectrophotometry at 280 nm.

### 2.7. Kd Value of Antibodies

Binding affinities of purified polyclonal and monoclonal antibodies were determined by ELISA-based equilibrium binding analysis. For polyclonal serum analyses, K*d* values were obtained using total serum antibodies (containing both IgM and IgG isotypes), as serum was not subjected to Protein G purification. In contrast, monoclonal antibodies were purified using Protein G and therefore represent IgG-only measurements. Briefly, plates were coated with 10 µg/mL peptide antigen, blocked with 2% (*w*/*v*) skim milk, and incubated with serial dilutions of antibody. Bound antibodies were detected using HRP-conjugated secondary antibodies, and absorbance was measured at 450 nm. Dissociation constants (K*d*) were calculated by nonlinear regression fitting using a one-site binding model. To ensure that K*d* values reflected intrinsic affinity rather than differences in antibody titer, binding analyses were performed using equal concentrations of purified antibodies. Because K*d* estimation is based on curve fitting across a concentration gradient, rather than on absolute absorbance values, the observed affinity gains are unlikely to result from variations in antibody concentration.

K*d* values were determined by nonlinear regression using GraphPad Prism 9 (GraphPad Software). Data were fitted to a one-site specific binding model:

$$Y=B_{\max}\times \frac{X}{Kd+X}$$

Fits with R^2^ < 0.95 were excluded and repeated. Equal concentrations of purified antibodies were used to minimize titer-dependent effects.

### 2.8. Statistical Analysis

All data are presented as mean ± standard deviation (SD). Statistical significance was assessed by Student’s *t*-test (two groups) or one-way ANOVA followed by Tukey’s post hoc test (multiple groups). A *p*-value < 0.05 was considered statistically significant.

## 3. Results

### 3.1. Systematic Triage Identifies Two Surface-Exposed Candidate Regions

This triage strategy was applied to ensure that selected peptide segments possess features that favor strong and specific antibody responses. Cross-species divergence helps avoid immune tolerance toward highly conserved self-like regions. Structural accessibility—particularly loop-like and solvent-exposed regions—maximizes the likelihood that antibodies raised against linear peptides will recognize the corresponding epitope on the native protein surface. Additionally, excluding segments with predicted post-translational modifications reduces the chance of targeting transient or structurally unstable motifs. Together, these principles increase the probability of identifying peptide fragments that are both immunogenic and functionally representative of the native protein. Using visfatin as a model antigen, cross-species alignment confirmed that the protein is highly conserved across mammals (Figure 2A). Direct comparison between human and mouse sequences revealed four locally divergent segments at residues 84–113, 298–327, 372–401, and 452–481 (Figure 2B). Structural mapping onto the visfatin crystal structure showed that all four regions adopt loop conformations (Figure 2C). However, the 372–401 segment is located within a buried region of the protein, and the 452–481 segment contains predicted phosphorylation sites. These two segments were therefore excluded from downstream peptide optimization. The remaining regions—residues 84–113 (V1) and 298–327 (V2)—were selected as candidate peptide epitopes for further mutational enhancement and immunogenicity testing (Table 1).

### 3.2. Conservative Substitutions Enhance Predicted MHC Binding

Despite deliberate selection of relatively divergent regions, human–mouse similarity remained high (84–113: 90% identity/100% similarity; 298–327: 93.3%/96.7%; Table 1). To enhance immunogenicity while preserving native features, we applied computational mutagenesis restricted to physicochemically similar residues (conservative substitutions), aiming to maintain local conformation (Figure 2D). IEDB predictions improved following single substitutions: H2-Kd binding rank for V1 (84–113) improved from 24.0% (V1-0) to 13.0% (F→Y; V1-1), and H2-IEd for V2 (298–327) from 8.35% (V2-0) to 1.31% (T→M; V2-1) (Table 2).

### 3.3. Mutant Peptides Elicit Higher Titers and Isotype Maturation

**Figure 3:**
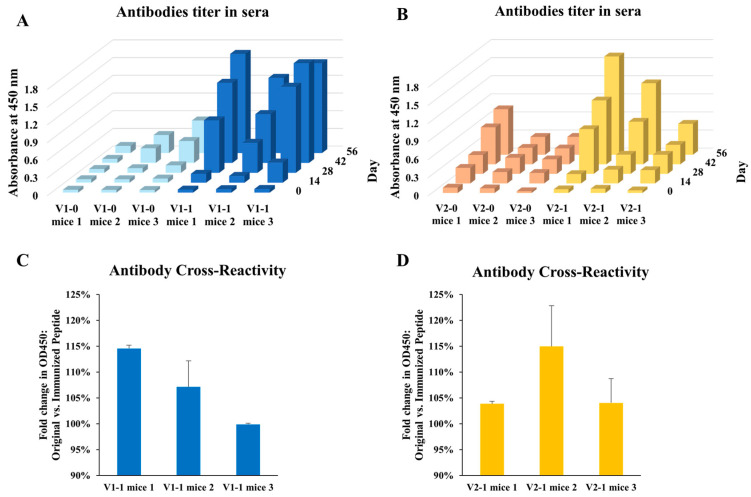
**Antibody responses elicited by wild-type and mutant visfatin peptides and their cross-reactivity with original antigens.** (**A**,**B**) Serum antibody titers measured by ELISA in mice immunized with wild-type (V1-0, V2-0) or mutant (V1-1, V2-1) peptides over a 56-day period. Mutant peptide groups showed higher absorbance at 450 nm compared with wild-type groups, indicating enhanced antibody responses. (**C**,**D**) Cross-reactivity of sera from mutant-immunized mice against wild-type peptides, expressed as fold change was calculated relative to OD450 values obtained from wild-type sera binding to the corresponding wild-type peptide. Antibodies induced by mutant peptides retained detectable binding to the corresponding wild-type epitopes, indicating preserved cross-recognition. Data represent individual mice (*n* = 3 per group); error bars indicate standard deviation.

**Figure 4:**
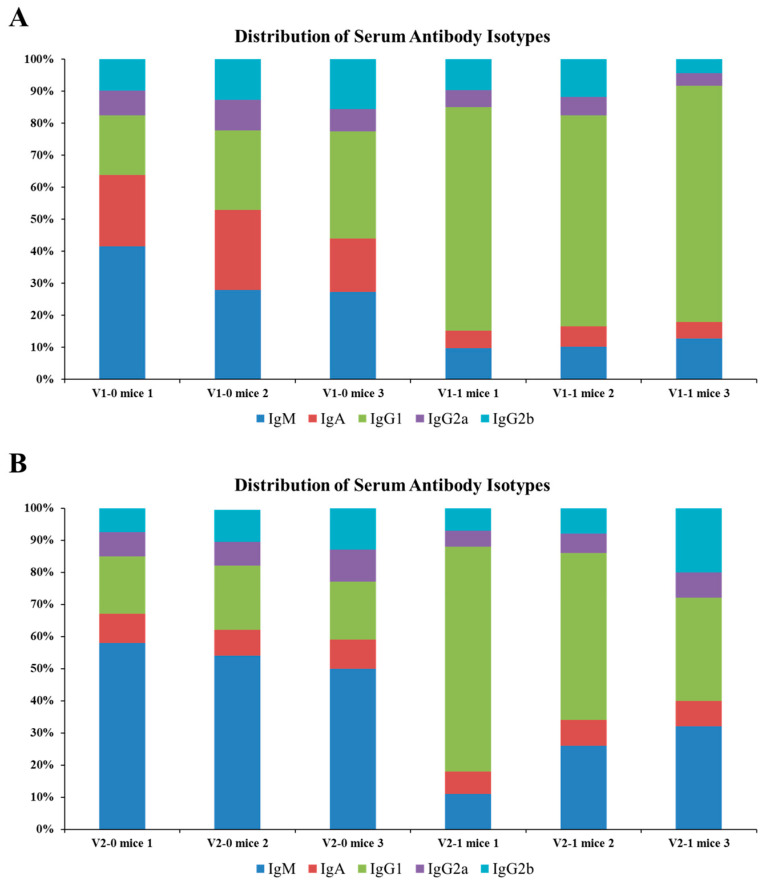
**Distribution of serum antibody isotypes in mice immunized with wild-type or mutant visfatin peptides.** Isotype distribution (IgM, IgA, IgG1, IgG2a, IgG2b) was measured by ELISA using serum samples collected on day 56 post-immunization. (**A**) Antibody isotype profiles in mice immunized with the wild-type (V1-0) and mutant (V1-1) peptides corresponding to the visfatin 84–113 region. (**B**) Antibody isotype profiles in mice immunized with the wild-type (V2-0) and mutant (V2-1) peptides corresponding to the visfatin 298–327 region. Mutant-peptide-immunized groups showed a higher proportion of IgG1 relative to IgM, indicating enhanced isotype switching and a more mature humoral response compared with wild-type groups. Each stacked bar represents an individual mouse (*n* = 3 per group). Absolute OD450 values for each isotype are provided in Supplementary Figure S1.

In mice, mutant peptides elicited higher ELISA titers than wild-type counterparts at days 42 and 56 (e.g., V1-1 mice 1 and V2-1 mice 1 OD450 ~1.8; Figure 3A,B). Sera from mutant-immunized animals also recognized the corresponding wild-type peptides (Figure 3C,D). Isotype profiling revealed a shift from IgM-dominant responses in wild-type groups toward increased IgG1 in mutant groups (Figure 4), consistent with isotype maturation and enhanced T-helper involvement (Th2-biased humoral response). These findings support the relevance of the sequence-level mutagenesis strategy and its potential for generating durable, application-oriented antibodies.

### 3.4. Affinity Gains with Preserved Binding to Wild-Type Peptides

**Figure 5:**
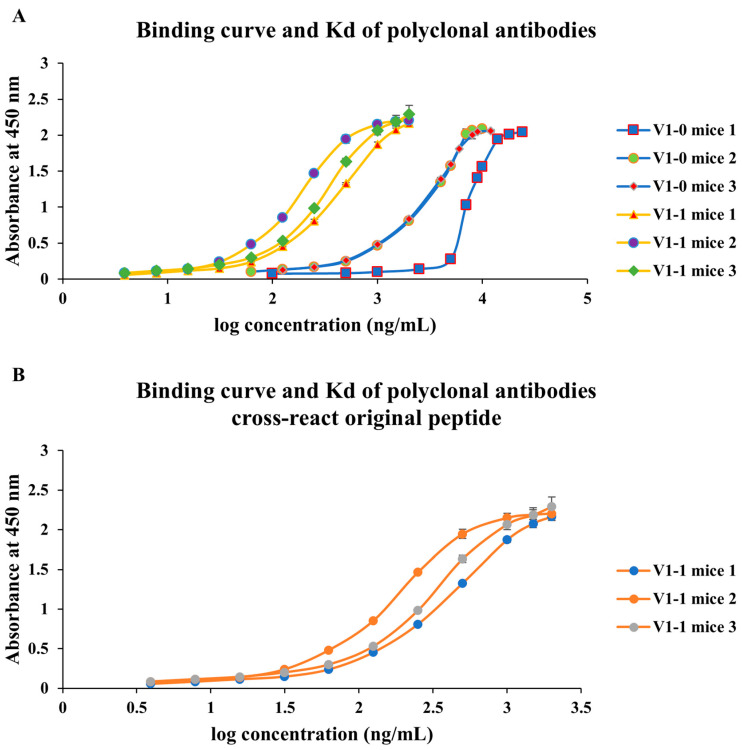
**Binding affinity of polyclonal antibodies elicited by wild-type and mutant peptides.** Serial dilutions of serum antibodies (X-axis: log antibody concentration) were incubated with plate-bound peptides to determine equilibrium binding curves and K*d* values. (**A**) Binding curves of sera from mice immunized with wild-type (V1-0) and mutant (V1-1) peptides, showing enhanced binding affinity in mutant-immunized groups. (**B**) Cross-reactivity of sera obtained from mutant-(V1-1)-immunized mice when tested against wild-type peptides, indicating that antibodies maintained detectable binding affinity to the original epitope. Data represent individual mice (*n* = 3 per group).

**Figure 6:**
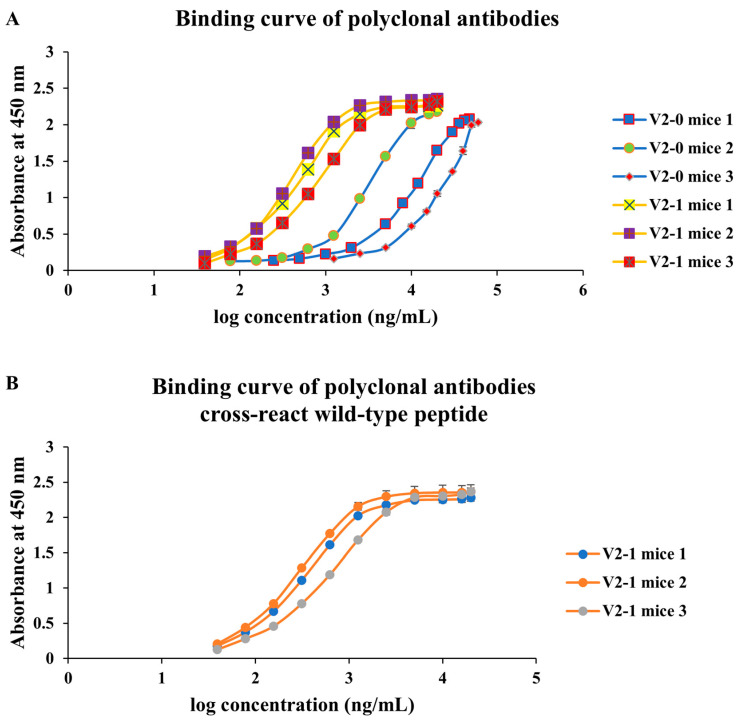
**Binding affinity and cross-reactivity of polyclonal antibodies elicited by visfatin V2 peptides.** Serial dilutions of serum antibodies (X-axis: log antibody concentration) were incubated with plate-bound peptides to determine equilibrium binding curves and K*d* values. (**A**) Binding curves of sera from mice immunized with the wild-type peptide (V2-0) or the mutant peptide (V2-1), showing that, compared to wild-type controls, mutant-immunized mice developed antibodies with higher binding affinities. (**B**) Cross-reactivity of sera from mutant (V2-1) immunized mice against the corresponding wild-type peptide, indicating that antibodies retained measurable recognition of the original epitope. Data represent individual mice (*n* = 3 per group).

**Figure 7:**
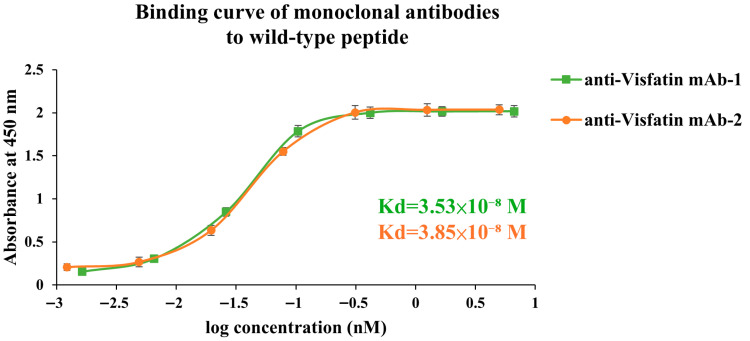
**Binding affinity of monoclonal antibodies derived from V1-1 hybridomas toward the wild-type visfatin peptide.** Binding curves of two anti-visfatin monoclonal antibodies (mAb-1 and mAb-2; both IgG1 isotype) measured by ELISA against the wild-type peptide (V1-0). Both antibodies were obtained from hybridoma clones generated in mice immunized with the V1-1 peptide and exhibited nanomolar binding activity, with dissociation constants (K*d*) of 3.53 × 10^−8^ M (mAb-1) and 3.85 × 10^−8^ M (mAb-2), confirming their ability to recognize the original visfatin epitope with comparable affinities.

Equilibrium binding analyses showed that polyclonal K*d* values generally improved from approximately 10^−8^ M (wild-type immunization) to the 10^−9^ M range (mutant immunization), indicating higher apparent affinity (Figure 5 and Figure 6). Importantly, sera from mutant groups retained detectable—and often comparable—binding to wild-type peptides, consistent with cross-reactive potential (Figure 5B and Figure 6B). Additionally, two anti-visfatin monoclonal antibodies demonstrated measurable binding to the wild-type peptide (3.53 × 10^−8^ M and 3.85 × 10^−8^ M; Figure 7), further supporting preservation of native-epitope recognition. Because polyclonal K*d* values were obtained from total serum, the apparent affinities may partially reflect IgM-mediated avidity. However, the monoclonal IgG1 K*d* values fall within the same range as the serum-derived curves, indicating that the observed affinity trend is consistent with true IgG binding. Notably, sera from wild-type-immunized mice were predominantly IgM, whereas mutant peptides induced IgG1-dominant responses, whose K*d* values more closely reflect intrinsic monovalent affinity. Thus, the actual affinity improvement resulting from sequence-level optimization is likely greater than what is measured from serum. Moreover, all K*d* determinations were performed using equal concentrations of purified antibodies, minimizing the influence of antibody titer. Because K*d* estimation is derived from curve fitting across concentration gradients rather than absolute absorbance values, the observed affinity enhancement represents genuine sequence-driven effects rather than concentration artifacts.

### 3.5. Double Substitutions Probe the Basis of Cross-Reactivity

**Figure 8:**
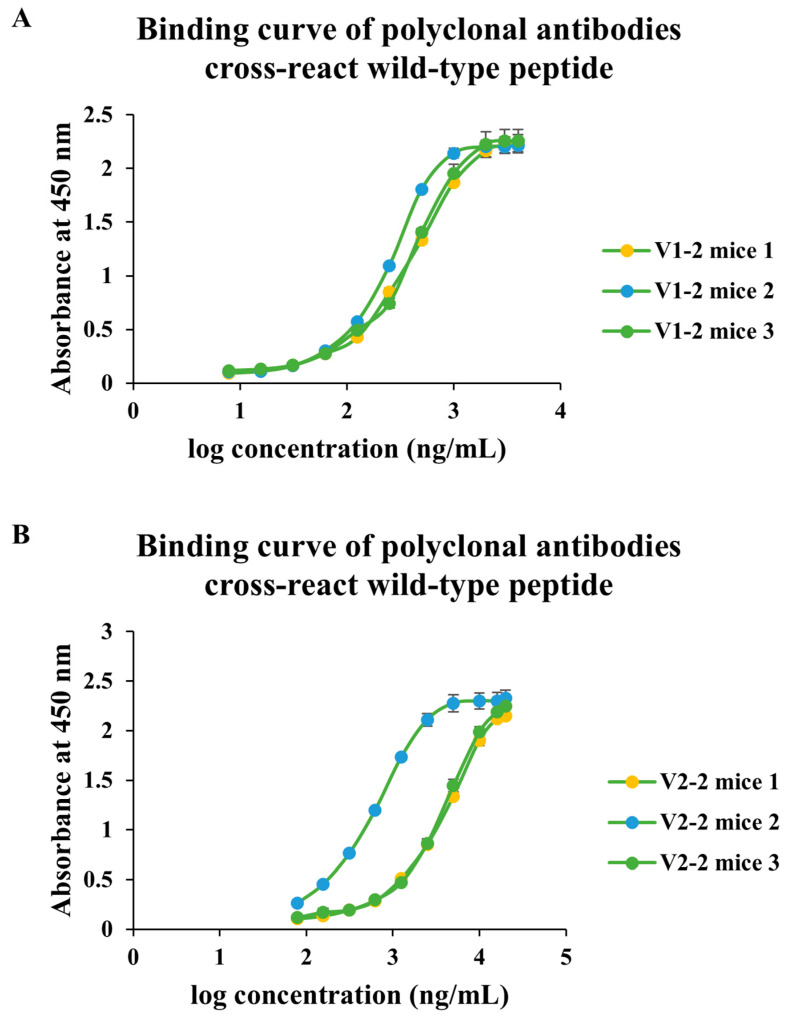
**Cross-reactivity of antibodies induced by double-mutant peptides.** (**A**) Binding curves of sera from mice immunized with the V1-2 double-mutant peptide against the corresponding wild-type peptide, showing that antibodies retained detectable recognition across all three animals. (**B**) Binding curves of sera from mice immunized with the V2-2 double-mutant peptide against the wild-type peptide, similarly demonstrating measurable cross-reactivity. Data represent individual mice (*n* = 3 per group).

To evaluate whether cross-recognition arose from antibodies targeting non-mutated regions or from insufficient perturbation by single substitutions, we designed double mutants with distinct spatial arrangements (Table 3): V1-2 incorporated distantly spaced substitutions, whereas V2-2 contained proximal substitutions. Both designs preserved recognition of the wild-type peptide with measurable affinity (Figure 8), indicating that substitution geometry can modulate binding strength while maintaining wild-type recognition. These results suggest that cross-reactivity is reproducible under different mutational configurations and likely reflects an intrinsic property of conservative sequence optimization rather than an incidental effect of a single design.

### 3.6. Portability to Additional Antigens

To assess generalizability, we extended the workflow to THBS2, ANTXR1, and IFITM1—targets with high human–mouse homology. In each case, sera recognized the corresponding wild-type peptides (Table 4), indicating that minimal, rational sequence edits coupled with MHC-guided prioritization constitute a portable strategy for peptide immunogenicity optimization.

Across independent MHC-I and MHC-II designs, conservative substitutions enhanced predicted MHC engagement and translated into higher antibody titers, isotype maturation, and modest affinity gains, while maintaining recognition of the native sequences. The double-mutation analysis further shows that spatial placement can fine-tune epitope recognition without abolishing wild-type binding, reinforcing the mechanistic plausibility of cross-reactivity within this sequence-level framework. Collectively, these findings validate the workflow’s applicability across structurally unrelated antigens, underscoring its potential as a generalizable tool for both immunological studies and antibody engineering.

## 4. Discussion

In summary, this study establishes a practical, sequence-level workflow that enhances peptide immunogenicity through minimal, conservative substitutions while preserving recognition of native epitopes. This conceptually bridges T-cell-oriented heteroclitic design with B-cell antibody engineering, demonstrating that sequence-level optimization can be extended beyond T-cell epitopes to guide rational antibody generation. Independent testing of MHC-I and MHC-II designs confirmed that enhanced responses were intrinsic to sequence optimization rather than formulation effects. Portability across additional antigens further supports this approach as a low-barrier route for generating application-ready, epitope-directed antibodies when commercial tools are unavailable.

Classical strategies to improve peptide immunogenicity—such as carrier conjugation (e.g., KLH, OVA, BSA) [10,11], adjuvant-intensive formulations (alum, CFA, TLR agonists) [15,16], or long/overlapping peptides [17,18]—can elevate antibody titers but often bias immune responses toward carrier epitopes, increase formulation complexity, or fail to address intrinsic sequence limitations. More recent approaches, including multi-epitope fusions and nanoparticle, liposomal [19], or virus-like particle (VLP) displays [20], enhance antigen density and trafficking but rely on specialized materials and infrastructure, reducing their accessibility for routine mechanistic studies. In contrast, our strategy focuses on intrinsic sequence optimization, offering a streamlined and formulation-independent alternative.

Conservative heteroclitic substitution design, which introduces rational amino-acid substitutions to strengthen MHC binding, has been extensively explored in T-cell epitope contexts to overcome immune tolerance while maintaining recognition of native antigens [13,21,22,23]. In contrast, applications to B-cell or antibody epitopes remain comparatively limited and often rely on engineered presentation systems—such as cyclic HER-2 peptides stabilized by disulfide bonds or nanoparticle-displayed dengue epitopes—to boost immunogenicity [24,25]. These strategies, while effective, require scaffold design, chemical cyclization, or nanoparticle formulation. Our work differs fundamentally by applying heteroclitic principles directly to linear peptides without modifying peptide topology or using specialized delivery platforms. The observed gains therefore arise from sequence-level optimization alone, independent of scaffold, carrier, or multivalent display. This platform-independent design lowers the technical threshold and makes the workflow accessible to laboratories without advanced materials or formulation capabilities, while still enabling predictable enhancement of peptide immunogenicity.

Operationally, we applied cross-species and structural triage to identify surface-exposed, PTM-free loop regions and independently prioritized one peptide for MHC class I and another for class II. Conservative point substitutions—restricted to physicochemically similar residues—were introduced and ranked using IEDB-based MHC binding predictions. In vivo*,* mutant peptides elicited higher antibody titers, exhibited isotype maturation (increased IgG1), and maintained measurable binding to wild-type sequences with generally improved affinity (K*d*), indicating preserved cross-recognition. Because sera from wild-type-immunized mice contained predominantly IgM, their apparent affinities may be influenced by avidity effects. In contrast, mutant peptides induced IgG1-dominant responses, whose measured K*d* more accurately reflects intrinsic monovalent affinity. This suggests that the true affinity gain produced by conservative substitutions is likely greater than what is captured by serum-based measurements. Consistency between serum trends and the two IgG1 monoclonal antibodies further supports that the affinity enhancement reflects genuine sequence-level optimization rather than multivalency or titer differences.

Two non-exclusive mechanisms may underline this phenomenon. First, conservative substitutions were intentionally restricted to physicochemically similar residues to minimize disruption of native B-cell epitope geometry. This design principle increases the likelihood that the mutant and wild-type peptides retain a shared structural motif, enabling recognition of both forms. This interpretation aligns with previous structural studies of heteroclitic T-cell peptides, which showed that subtle substitutions can stabilize peptide–MHC interactions without altering the overall epitope conformation. Such stabilization prolongs peptide presentation and promotes selection of immune clones recognizing shared structural motifs rather than individual residues, thereby explaining the retained cross-recognition of wild-type antigens [26]. Our findings parallel those observations by Chen et al. [26], who demonstrated that heteroclitic substitutions can stabilize MHC–peptide complexes and broaden immune recognition toward conserved structural features. Second, because our workflow favors extracellular or surface-exposed regions, endogenous wild-type epitopes in the host may continue to stimulate plasma cells, sustaining antibody production and memory responses [27,28,29].

Importantly, these two mechanisms remain testable hypotheses rather than firm conclusions. If structural preservation is the dominant mechanism, mutant- and wild-type-binding affinities should remain similar—an observation preliminarily reflected in our K*d* measurements. Conversely, if endogenous antigen exposure plays a major role, distinct cross-recognition patterns may emerge in visfatin-deficient or conditional knockout models. Although our small cohort size limits definitive mechanistic resolution, identifying these as open questions provides a balanced interpretation and outlines clear directions for future experimental validation.

Double-substitution experiments, arranged either distantly or proximally, further revealed that mutation placement can modulate binding strength without abolishing wild-type recognition. This suggests that immunogenicity can be fine-tuned through geometric design principles. Importantly, the observed improvements arise from minimal, rational sequence edits rather than from adjuvant or carrier effects, allowing the workflow to remain rapid, reproducible, and compatible with standard immunization protocols.

This proof-of-concept study has several limitations, including the small mouse cohorts (*n* = 3 per group) and a limited antigen panel. Larger-scale validation, orthogonal affinity assays (e.g., SPR or BLI), and fine epitope mapping would further strengthen the mechanistic interpretation. Future integration of structure-guided modeling, deep mutational scanning, or high-throughput screening could help codify substitution rules and automate peptide design. Additionally, combining sequence-level optimization with selected presentation platforms (e.g., nanoparticle-based carriers) may further enhance immunogenicity while maintaining recognition of native epitopes [30,31]. Finally, because all ELISA assays utilized full-length 30-mer peptides, the minimal linear epitope boundaries recognized by the resulting antibodies remain undefined. Although the antibodies bind the corresponding wild-type sequences, future work using truncated peptides or alanine scanning will be needed to determine the shortest recognition motif, which will be essential for applications such as Western blotting and linear-epitope assays.

While this work focuses on a research-oriented proof-of-concept, potential translational applications—such as diagnostic targets, neoantigens, or therapeutic antibody development—would require additional safety assessment. Although the conservative substitutions used here were intentionally restricted to physicochemically similar residues to preserve native epitope architecture, clinical translation would necessitate evaluating whether such modifications could inadvertently introduce cross-reactivity with unrelated self-antigens or structurally similar host peptides. Likewise, for pathogen-directed antibodies, it would be important to assess whether substitutions might narrow or shift recognition breadth toward naturally circulating viral variants. These considerations extend beyond the scope of the present study but represent important next steps for future preclinical validation.

## 5. Conclusions

In conclusion, our findings demonstrate that conservative, sequence-level mutagenesis provides a simple, reproducible, and formulation-independent strategy to enhance peptide immunogenicity while preserving recognition of native epitopes. This approach may facilitate antibody development targeting diagnostic markers, neoantigens, and conserved viral epitopes—particularly in settings where commercial antibodies are unavailable or insufficient. By bridging the gap between rational peptide design and accessible antibody generation, this workflow offers a practical and generalizable toolkit for immunological and translational research. The mechanistic explanations proposed here—structural preservation of the native epitope and potential boosting by endogenous antigen exposure—should be regarded as testable hypotheses rather than definitive mechanisms. Future studies incorporating structural mapping, peptide–MHC modeling, and validation in knockout models will be required to determine the dominant contributors to cross-reactivity. Moreover, the design principles described in this study can be readily adapted for neoantigen discovery, fine epitope mapping, and therapeutic antibody engineering. As this platform advances toward translational applications, evaluation in disease-relevant models (e.g., tumor-bearing or viral challenge systems) and assessment of potential safety concerns, including unintended cross-reactivity with host proteins or circulating pathogen variants, will be essential.

## Figures and Tables

**Table 1:** **Comparison of visfatin peptide sequences between human and mouse.** Pairwise alignment of human and mouse visfatin/NAMPT highlighting the regions selected for epitope design (aa 84–113 and 298–327). The table reports start–end positions, identity and similarity percentages, and residue differences at each position (variant residues highlighted). These data were used to nominate peptide segments with sufficient divergence for immunogenicity optimization while preserving structural context. N/A indicates that the item is not applicable.

Antigen	Species	Peptide Sequence (Wild-Type)	Location	Identity (%)	Similarity (%)
Visfatin	Human	KDVYKEHFQDDVFNEKGWNYILEKYDGHLP	84–113	N/A	N/A
Visfatin	Mouse	KEVYREHFQDDVFNERGWNYILEKYDGHLP	84–113	90	100
Visfatin	Human	LIVSRSTQAPLIIRPDSGNPLDTVLKVLEI	298–327	N/A	N/A
Visfatin	Mouse	LIVSRSTEAPLIIRPDSGNPLDTVLKVLDI	298–327	93.3	96.7

**Table 2:** **Visfatin peptide antigens and single-substitution variants used for immunization.** Sequence list of wild-type peptides and corresponding single-point mutants (e.g., V1-0/V1-1; V2-0/V2-1). Columns include peptide sequence (mutated residues indicated), annotation (wild-type vs. mutation and position), MHC haplotype evaluated, and IEDB-predicted binding percentile rank. Lower percentile rank indicates stronger predicted MHC binding. The arrow (→) indicates an amino acid substitution. Δ represents the difference between the mutant and the wild-type values (Mutant − WT).

Name	Peptide Sequence (Mutated Residues Highlighted in Red)	Annotation	MHC Haplotype	Percentile Rank (%, IEDB Prediction)	Δ Percentile Rank (Mutant − WT)
V1-0	KDVYKEHFQDDVFNEKGWNYILEKYDGHLP	Visfatin wild-type peptide (84–113)	H2-Kd	24	−11.0%
V1-1	KDVYKEHYQDDVFNEKGWNYILEKYDGHLP	Mutation sequence (F→Y)		13	
V2-0	LIVSRSTQAPLIIRPDSGNPLDTVLKVLEI	Visfatin wild-type peptide (298–327)	H2-IEd	8.35	−7.04%
V2-1	LIVSRSMQAPLIIRPDSGNPLDTVLKVLEI	Mutation sequence (T→M)		1.31	

**Table 3:** **Double-mutation designs for visfatin peptides and their predicted/experimental properties.** Sequences of the two-point mutants (V1-2 and V2-2) generated by conservative substitutions. V1-2 places substitutions at distant positions, whereas V2-2 places them proximally. The table summarizes sequence changes, positions, MHC haplotype, IEDB percentile rank, and assay readouts where available (e.g., cross-reactivity to the wild-type peptide and binding parameters). Δ represents the difference between the mutant and the wild-type values (Mutant − WT). The arrow (→) indicates an amino acid substitution.

Name	Peptide Sequence (Mutated Residues Highlighted in Red)	Annotation	MHC Haplotype	Percentile Rank (%, IEDB Prediction)	Δ Percentile Rank (Mutant − WT)	Recognized Wild-Type Peptide
V1-0	KDVYKEHFQDDVFNEKGWNYILEKYDGHLP	Visfatin wild-type peptide (84–113)	H2-Kd	24	−19.7%	Yes
V1-2	KDVYKEHYQDDVFNEKIWNYILEKYDGHLP	Mutation sequence (F→Y, G→I)		4.3		Yes
V2-0	LIVSRSTQAPLIIRPDSGNPLDTVLKVLEI	Visfatin wild-type peptide (298–327)	H2-IEd	8.35	−7.89%	Yes
V2-2	LIVMRSMQAPLIIRPDSGNPLDTVLKVLEI	Mutation sequence (S→M, T→M)		0.46		Yes

**Table 4:** **Extension of the peptide-engineering workflow to additional antigens.** Peptide antigens derived from THBS2, ANTXR1, and IFITM1 (human and mouse) were evaluated using the same triage and mutagenesis workflow applied to visfatin. The table lists species, peptide sequences (and corresponding mutant variants), residue locations within the parent proteins, and qualitative readouts of antigen recognition at the wild-type peptide levels. Across these three additional targets, sera exhibited measurable antibody titers and detectable binding to the corresponding wild-type peptides, illustrating that the workflow is portable across structurally unrelated antigens.

Antigen (Gene Symbol)	Species	Peptide Sequence (Wild-Type)	Location	Mutated Immunization Sequence (Red Indicates Mutation)	Human Peptide Recognition (Wild-Type)
THBS2 (Thrombospondin 2)	Human	CDLIGPVALDEPFYEHLQAEKSRMYVAKGSARES	167–200	CDLIDEFALDEPFYEHLQAEKSRMYVAKGSARES	Recognized
	Mouse	CDLIDSVTLEEPFYEQLEVDRSRMYVAKGASRES	167–200		
ANTXR1 (Anthrax toxin receptor 1)	Human	TDGELHEDLFFYSEREANRSRDLGAIVYCVGV	149–180	TDGELHEDLFFYSERNANRMRDLGAIVYCVGV	Recognized
	Mouse	TDGELHEDLFFYSEREANRSRDLGAIVYCVGV	145–176		
IFITM1 (Interferon Induced Transmembrane Protein 1)	Human	MHKEEHEVAVLGAPPSTILPRSTVINIHSETSVPDH	1–36	MHKEEHEVAVLGPPPSTILPRSTVINIHSETSVPDH	Recognized
	Mouse	MPKEQQEVVVLGSPHISTSATATTINM-PEISTPDH	1–36		

## Data Availability

The original contributions presented in this study are included in the article/Supplementary Material. Further inquiries can be directed to the corresponding authors.

## Supplementary Materials

The following are available online at https://www.mdpi.com/article/10.3390/antib14040106/s1, Figure S1: Absolute OD450 values of individual antibody isotypes in V1 and V2 groups, Table S1: Complete amino acid sequences of all peptide variants experimentally tested in this study.

## Abbreviations

**ANTXR1**: anthrax toxin receptor 1; **CFA**: complete Freund’s adjuvant; **IEDB**: Immune Epitope Database; **IFITM1**: interferon-induced transmembrane protein 1; **NAMPT** (visfatin): nicotinamide phosphoribosyltransferase (visfatin); **PTM**: post-translational modification; **THBS2**: thrombospondin 2; **VLP**: virus-like particle; **WT**: wild type
