# Supplementary material for: A Reproducible Sequence-Level Strategy to Enhance Peptide Immunogenicity While Preserving Wild-Type Epitope Recognition
Source: Antibodies (Basel). 2025 Dec 12;14(4):106. doi: 10.3390/antib14040106 (PMC12730111; doi:10.3390/antib14040106)
Supplement: Supplementary file 1 [file antibodies-14-00106-s001.zip › antibodies-4019008-supplementary.pdf]

Supplementary data

Name	Peptide sequence
V1-0	KDVYKEHFQDDVFNEKGWNYILEKYDGHLP
V1-1	KDVYKEHYQDDVFNEKGWNYILEKYDGHLP
V1-2	KDVYKEHYQDDVFNEKIWNYILEKYDGHLP
V2-0	LIVSRSTQAPLIIRPDSGNPLDTVLKVLEI
V2-1	LIVSRSMQAPLIIRPDSGNPLDTVLKVLEI
V2-2	LIVMRSMQAPLIIRPDSGNPLDTVLKVLEI

Table S1. Complete amino acid sequences of all peptide variants experimentally tested in this study. This table lists the full sequences of the six peptide variants evaluated in the immunization experiments, including the wild-type segments (V1-0 and V2-0) and their single- and double-mutant derivatives (V1-1, V1-2, V2-1, V2-2). Mutation sites and residue substitutions are annotated in red for each variant, allowing direct visualization of the amino-acid changes introduced during peptide engineering. Peptide candidates removed during preliminary triage were not included because they were not experimentally tested. This table enhances transparency and supports reproducibility of the peptide design workflow.

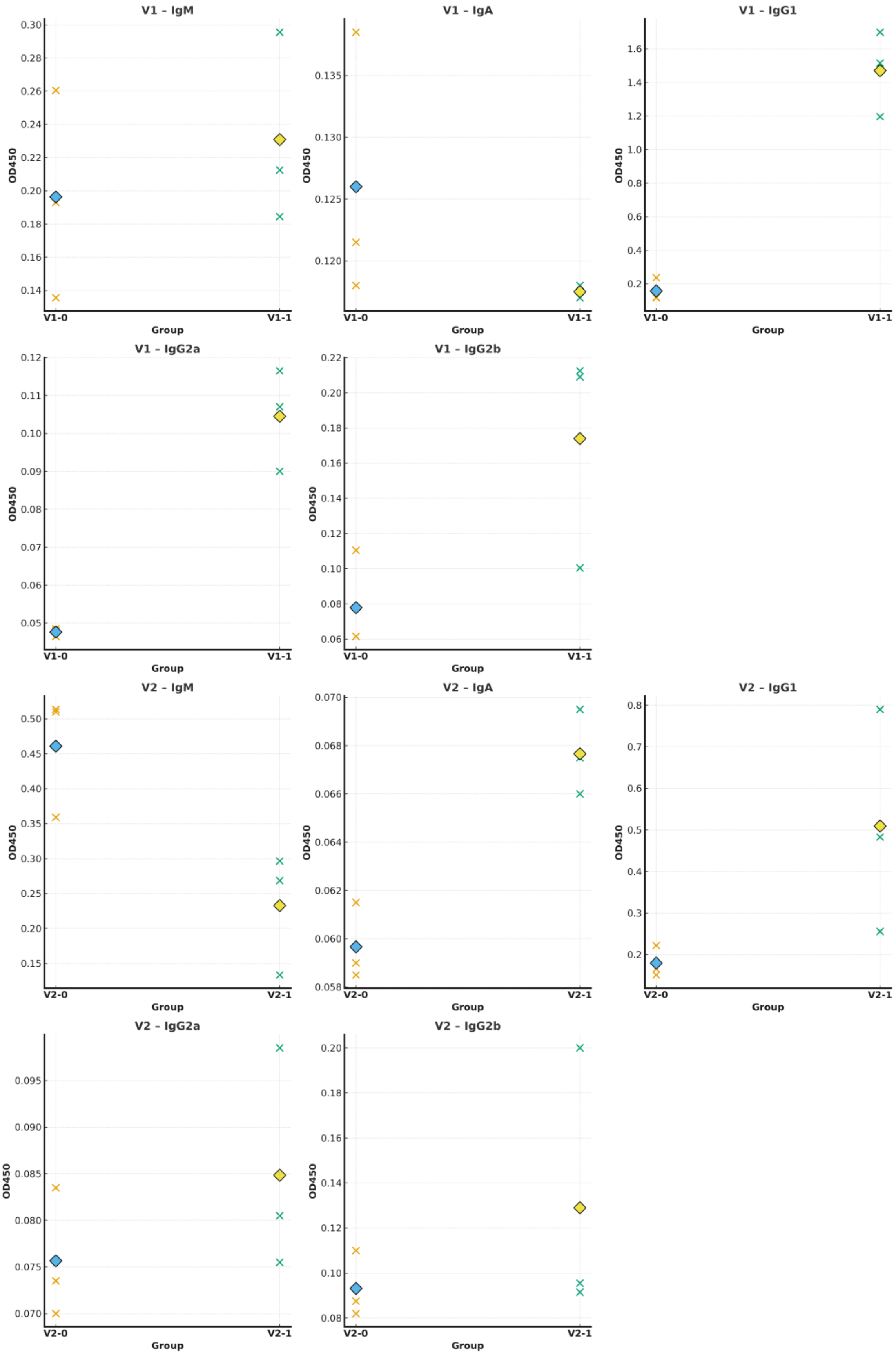

Figure S1. Absolute OD450 values of individual antibody isotypes in V1 and V2 groups. For each isotype, scatter points represent measurements from individual mice

(n = 3 per group), and black-edged diamond symbols indicate group means. Panels are arranged to show V1 isotypes (top two rows) followed by V2 isotypes (bottom two rows). These data provide the raw isotype composition underlying the relative-class distributions shown in Figure 4 and allow assessment of whether total antibody output differs between wild-type and mutant peptide groups.
